# Effects of Conventional Exercises on Lower Back Pain and/or Pelvic Girdle Pain in Pregnancy: A Systematic Review and Meta-Analysis

**DOI:** 10.7759/cureus.42010

**Published:** 2023-07-17

**Authors:** Madhuri Kandru, Sri Nikhil Zallipalli, Nikith Kashyap Dendukuri, Saichand Linga, Loshini Jeewa, Ashvini Jeewa, Sher Bahadur Sunar

**Affiliations:** 1 Department of Obstetrics and Gynaecology, Bharati Vidyapeeth Deemed University Medical College and Hospital, Sangli, IND; 2 Department of Trauma and Orthopaedics, Royal National Orthopaedic Hospital NHS Trust, London, GBR; 3 Institute of Orthopaedics and Musculoskeletal Science, Royal National Orthopaedic Hospital (RNOH) Campus, University College London (UCL), London, GBR; 4 General Practice, Ashley Medical Practice, Walton-on-Thames, GBR; 5 Department of Trauma and Orthopaedics, South Tyneside and Sunderland NHS Foundation Trust, South Shields, GBR; 6 Department of Internal Medicine, Chester Medical School, University of Chester, Chester, GBR; 7 Department of Internal Medicine, Countess of Chester Hospital NHS Foundation Trust, Chester, GBR

**Keywords:** vas scores, meta-analysis, exercise, pregnancy, pelvic girdle pain, low back pain

## Abstract

Pregnant women frequently complain of low back discomfort associated with their pregnancies. On their quality of life, it could have a detrimental effect. Pregnancy-related low back pain (LBP) and pelvic girdle pain (PGP) are associated with substantial direct and indirect expenditures. Evidence addressing strategies to treat and prevent these illnesses needs to be clarified. This review aimed to examine the connection between exercise, LBP, and PGP. To find relevant studies (in the English language) that matched the inclusion and exclusion criteria, a systematic search of peer-reviewed literature was carried out using the Cochrane Database of Systematic Reviews, the Cochrane Central Register of Controlled Trials, Scopus, the Web of Science, Pub Med, and ClinicalTrials.Gov. The publishing window was limited to the previous 10 years (2012-2022). Utilizing Review Manager version 5.4 (The Nordic Cochrane Centre, The Cochrane Collaboration, Copenhagen), the results were examined. JADAD ratings were used to evaluate the quality of the included studies. To analyze the endpoints, the mean, standard mean difference (SMD), and 95% confidence intervals (CI) were determined. We chose 16 randomized controlled trials (RCTs) that included 1885 pregnant individuals with pelvic girdle and/or lower back discomfort. The combined data showed that the exercise group had lower VAS scores than the control group. The final result, however, did not significantly differ. Most of the studies had high JADAD scores, ranging from 3 to 5 points. Lower back pain and/or pelvic girdle discomfort during pregnancy are not influenced by exercise; however, women who are provided with a regular exercise program appear to manage the condition effectively with improved functional status.

## Introduction and background

**Figure 1:**
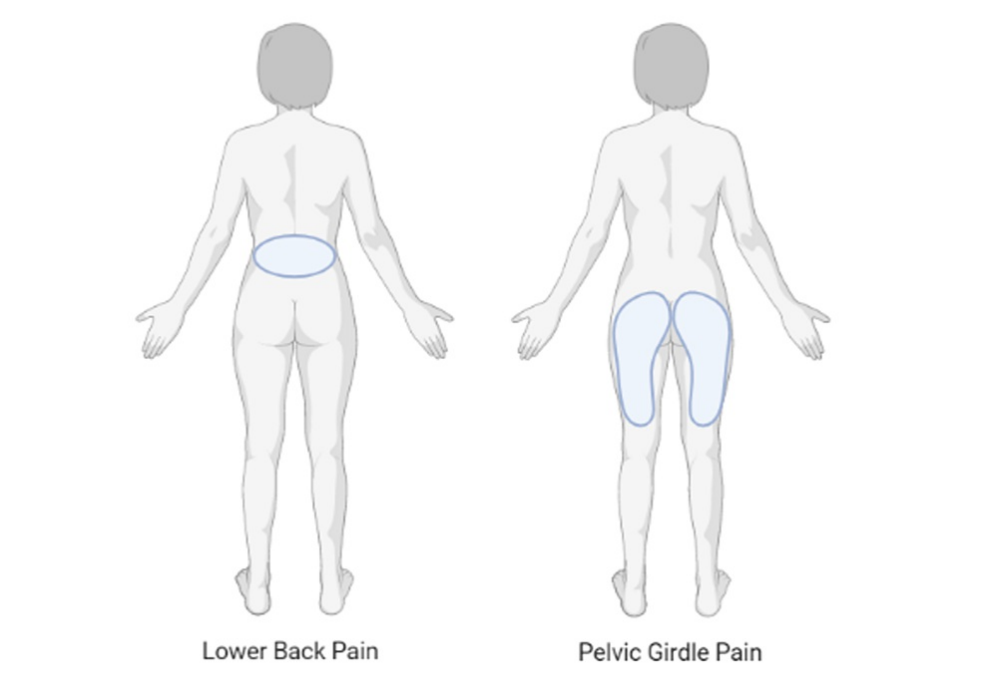
Pictorial representation of lower back pain and pelvic girdle pain positions.

Pregnant women frequently have lower back pain (LBP) and/or pelvic girdle pain (PGP), which has a significant negative influence on their quality of life. About a third of people with LBP and/or PGP have significant pain, which is frequently accompanied by restrictions on a woman's capacity for productive employment and results in a poor quality of life [1]. As a result, the woman is less productive on an individual level in her daily routine duties. Pregnancy is the time when many LBP and/or PGP sufferers first have an event [2]. Given the incapacitating consequences of LBP during pregnancy, it is frequently left untreated and is seen by women as a natural and unavoidable aspect of pregnancy. The precise origin of LBP during pregnancy is unclear; it is frequently thought to be complex in nature and linked to changes in biomechanics, vascular structure, and hormone levels [3]. The prevalence of LBP during pregnancy varies between 25% and 90%, with the majority of study results projecting that 50% of pregnant women will experience LBP [4]. They will have a lower quality of life since one-third of them will experience severe discomfort. Around 80% of women with LBP claim it interferes with their everyday activities, and 10% say they are incapable of working. Pregnant women will experience PGP in 20% of cases. According to a study conducted in the Netherlands regarding PGP, 38% of women still experience symptoms three months after giving birth and 13.8% after twelve months [5,6]. Elevated pain intensity during pregnancy has been linked to worse postpartum recovery rates. Pain in the pubic symphysis and both sacroiliac joints may be regarded as severe PGP (Figure 1) [7]. Several approaches can be used to treat pregnancy-related pain, such as land-based or aquatic exercises, pelvic belts, manual therapy (osteopathic manipulative therapy, spinal manipulative therapy, neuro-emotional technique, craniosacral therapy), "transcutaneous electrical nerve stimulation", "Kinesio taping", yoga, acupuncture, acupuncture plus exercises, and an integrated strategy combining physical therapy, sporting activities, and education [4,8].

In addressing LBP during pregnancy, several investigations in diverse populations have been carried out. The topic is still debatable, and the etiology still needs to be well known [1]. While there are several proposed reasons for the pathophysiology of LBP during the prenatal period, their scientific foundations still need to be established. Additionally linked are hormonal changes, which raise the danger of joint and nerve damage. One of the reasons for pelvic discomfort during pregnancy is recognized to be elevated loosening of joints, which is brought on by heightened levels of relaxin, progesterone, and estrogen [9]. The most often proposed explanations are linked to mechanical variables brought on by weight growth during pregnancy, which increases belly sagittal diameter and causes a corresponding anterior shift of the body's gravity center, putting stress on the lower back [2]. It appears that sacroiliac asymmetries are widespread. Clinically substantial improvements in function and asymmetries suggest that SIFFT may be a helpful assessment tool for prescribing a straightforward self-directed remedial exercise [10]. Findings imply that issues with the pubic symphysis are related to an anterior displacement. To counteract this anterior shift, postural adjustments may be made, which would lead to bending and put more strain on the lower back [11]. It has been proposed that LBP and PFD (pelvic floor dysfunction) are related. A good conjunction with a negative Active Straight Leg Raise (ASLR) test may indicate that the pelvic floor muscles are working harder to make up for the weak pelvic equilibrium [12].

Pregnancy-related low back discomfort has a significant impact on women's lives. The most frequent reason for sick time following birth is low back discomfort [13]. Early detection and treatment will result in the greatest results, taking into account the uniqueness of each woman and pregnancy. The gold standard for treatment is traditional management, which includes physiotherapy, stabilization belts, electrical stimulation of the nerves, medication, acupuncture, massage therapy, rest, and yoga [14]. Pregnancy-related LBP and/or PGP often have a positive prognosis if they are identified and treated early after pregnancy. It is generally known that using analgesics while pregnant has health hazards [15]. For instance, opioids are not regarded as appropriate for use during pregnancy, and non-steroidal anti-inflammatory drugs (NSAIDs) are not recommended throughout the third trimester [16]. As a result, taking medications to treat LBP while pregnant has not proven effective. Because of this, many women view LBP as a typical discomfort they must endure while pregnant. Nevertheless, non-pharmacological therapies, frequently delivered by physiotherapists, have been reported to be successful in controlling LBP during pregnancy. These include soft tissue massage, posture instruction, stabilization exercises, and electrical nerve stimulation through the skin [17].

Recent systematic reviews and meta-analyses have incorporated trials on both the primary and secondary management of LBP and PGP. Nevertheless, it is not apparent if advantages apply to LBP and PGP through first-line prevention. The current systematic review and meta-analysis of randomized controlled trials and observational controlled studies' effects of exercise on the main means of avoiding LBP and PGP in pregnancy were conducted. The purpose of this review is to emphasize particular treatment suggestions by conducting a systematic review and meta-analysis of the relevant studies and providing their clinical findings for the treatment of pregnancy-related back pain.

## Review

Methodology

**Figure 2:**
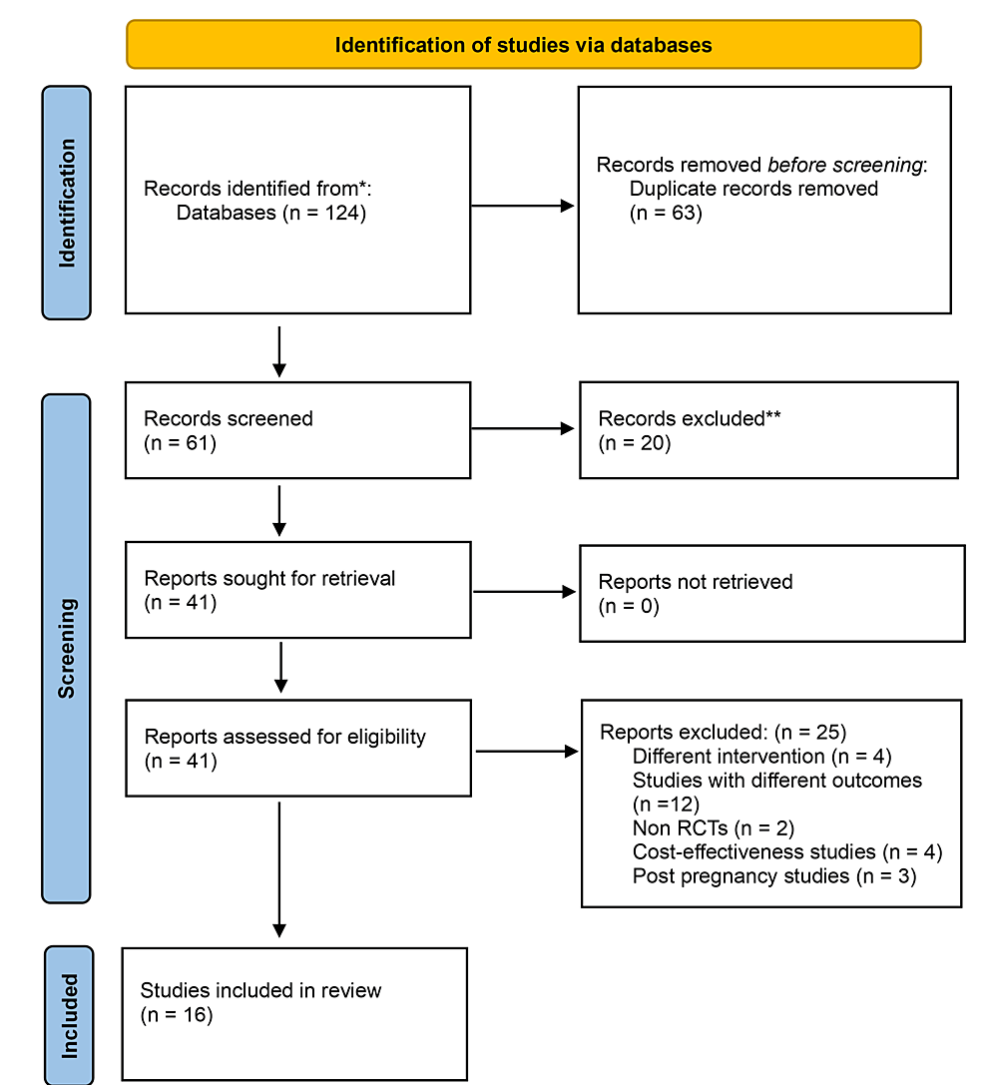
PRISMA flow diagram illustration of the search strategy and selection of relevant articles for the qualitative analysis.

The "Preferred Reporting Items for Systematic Reviews and Meta-Analyses" (PRISMA) statement and checklist were used to guide this systematic review and meta-analysis [18]. A PRISMA flow diagram is shown in Figure 2 to illustrate the main procedures engaged in the search process. The entire extraction of records was examined using the context of the chosen keywords to spot any publications that may have been collected from multiple sources twice. The remaining articles were then assessed in full text for eligibility to find any that met the inclusion criteria.

Eligibility Criteria

This study followed the "participants," "interventions," "comparisons," "outcomes," and "study design" (PICOS) framework.

Population

The population of interest was pregnant women with lower back pain and/or pelvic girdle pain. Studies included pregnant women at any stage of pregnancy who were at risk of developing, or already had, LBP, PGP, or the two conditions together as claimed symptoms-wise by the mothers, or as determined by doctors after performing appropriate investigations.

Intervention

Prenatal exercise of any degree of intensity, length of time, capacity, or kind was the intervention, and it may have been monitored quantitatively or qualitatively. Exercise during pregnancy can be acute (a single exercise event) or chronic (a regular activity). Exercise-only interventions or exercises coupled with additional interventions (such as nutrition or music, referred to as "exercise + co-interventions") were both taken into consideration. Trials that started exercising after the start of labor were not qualified. Even though exercise is a form of bodily activity, the words are used synonymously throughout this evaluation. Any skeletal muscle-driven action that causes the consumption of energy above the resting state is referred to as exercise or physical activity.

Comparison

The exercise group is compared to the usual activity group or no activity group. Exercise with a different frequency, intensity, time frame, magnitude, or kind, a different length of treatment, or activity in various trimesters were all acceptable comparators.

Outcome

Relevant outcomes were the effects and severity of LBP and PGP during pregnancy before and after treatment. The effectiveness of the therapy as rated by the women themselves, the symptom reduction, involvement in routine daily activities, and adverse effects assessed at the conclusion of treatment while pregnant were all included as outcomes. Pain severity (VAS scores), functional status (ability to conduct daily activities), days missed from work due to illness, and adverse consequences for pregnant women, as determined by the trialist, were also included.

Study Design

Randomized controlled trials and observational controlled trials were included.

Search Strategy

A comprehensive search was created and run in the following databases: Cochrane Database of Systematic Reviews, Cochrane Central Register of Controlled Trials, Scopus, Web of Science, PubMed, and ClinicalTrials. Gov. using the following search strategy: (pregnancy) AND (exercise) AND (lower back pain) AND (pelvic girdle pain) OR (pelvic girdle pain) OR (lower back pain). Studies published in the English language were retrieved. The publication year was restricted to the last 10 years (January 2012 to December 2022) to obtain recent evidence.

Study Selection and Data Extraction

Studies found using search techniques were transferred to Endnote and Review Manager software for data extraction and de-duplication before being taken into account as a single review. Two reviewers separately examined the titles and abstracts of all the extracted publications. The entire text of abstracts that at least one reviewer determined to have satisfied the inclusion requirements was automatically obtained. On January 22, 2023, two reviewers independently evaluated full-text publications for the applicable demographics. A second review was done for studies where at least one reviewer had suggested exclusion before a final exclusion determination was made. A third reviewer was consulted in the event that a disagreement arose that could not be settled through discussions. In situations where there were multiple studies underway, the most up-to-date or comprehensive publication was chosen as the "parent" study; nonetheless, appropriate information was taken from all articles. Study characteristics (such as author, year, and study design) as well as demographics (such as participant count, age, pregnancy status, and pregnancy complications) as well as intervention-related information (such as prescribed and/or measured physical activity rate, level of difficulty, time, kind, and capacity as well as time frame and assessment tool) and outcomes (such as incidence and/or degree of LBP and PGP) were all retrieved from the articles. We contacted the respective authors of the studies to see if any missing data were required; however, the approach was unsuccessful.

Quality of Evidence Assessment

The degree of confidence in the effects that have been observed is decreased by variables such as indirectness, discrepancy, imprecision, and the likelihood of publication bias; hence, information from randomized controlled trials (RCTs) was classified as "low" quality if these issues were present. In particular, the Cochrane risk of bias tool (ROB tool) [19] was used to evaluate the risk of bias in RCTs. These assessments are systematic reviews that were done in accordance with prior health behavior recommendations [20]. All studies were examined for any potential biases, including selection bias (randomization and allocation concealment), reporting bias (selective/incomplete outcome reporting), performance bias (double blinding), detection bias (faulty outcome measurement), attrition bias (incomplete follow-up, high loss to follow-up), and "other" sources of bias. Given the objective nature of physical exercise treatments, it is impossible to blind participants to group allocation; therefore, the selection risk of bias was graded as "low" if this was the sole cause of bias found. When 10% of the study participants left the trial for any rationality, the purpose of treatment evaluation was not employed, and performance bias was evaluated as "strong." When heterogeneity was substantial (I2 ≥ 50%), it was deemed problematic when there was a discrepancy between trials. Publication bias was not graded lower and was considered non-estimable if there were fewer than 10 publications. The GRADE tables were examined by an additional author as an assurance of quality, and one reviewer assessed the quality of the data.

Statistical Analysis

Utilizing Review Manager 5.4.1 (The Nordic Cochrane Centre, The Cochrane Collaboration, Copenhagen), data were analyzed. The articles that were shortlisted were combined and weighted. The mean (M), standard mean difference (SMD), and 95% confidence interval (CI) were calculated for both fixed-effects and random-effects models. Heterogeneity was examined with the χ2 test and quantified with the I2 statistic. A random-effects model was used if there was heterogeneity (P=0.01 or I2>50%) between the trials; otherwise, a fixed-effects model was employed. The random-effects model was used for the meta-analyses.

Results

Study Characteristics

The PRISMA guidelines led to the retrieval of 124 relevant research studies. The article titles and abstracts were examined to filter out publications that were not appropriate. After reading the whole text of the studies that met the requirements, we disregarded those that did not. Finally, 16 studies covering 1885 patients were included in our meta-analysis, as shown in Figure 2. One thousand eight hundred and eighty-five patients were divided into two groups: the exercise group received 960 (50.93%) patients, while the control group received 925 (49.07%) patients (Table 1). The studies considered in this meta-analysis were all RCTs. Table 1 displays the main features of the studies that were included as well as the physical function and pain baseline ratings. Only one research study was an observational controlled study, and the majority of randomized controlled trials utilized a parallel-group design. Despite some of the baseline parameters (such as the specifics of the program of study and the length of the follow-up) (Table 1) differing among studies as a whole, the initial factors were generally consistent across the studies that were included.

**Table 1:** Characteristics of the studies included in the analysis. RCT: randomized controlled trial; SD: standard deviation; OR: odds ratio [21-36].

Author	Study design	Participants		Follow-up	Pain scores (baseline) mean (SD)		Pain scores (after follow-up) mean (SD)	
		Exercise group	Control group		Exercise group	Control group	Exercise group	Control group
Abu et al. [21]	RCT	73	72	6 weeks	4.83 (1.19)	4.66 (1.33)	3.3 (0.81)	4.66 (1.33)
Akmese and Oran [22]	RCT	33	33	2 months	7.78 (1.61)	7.69 (1.75)	3.72 (1.25)	9.03 (0.98)
Eggen et al. [23]	RCT	52	54	16 weeks	0.5 (0.4)	0.5 (0.3)	1.7 (1.4)	1.7 (1.3)
Field et al. [24]	RCT	28	28	12 weeks	4.17 (2.26)	4.85 (2.43)	2.75 (2.19)	5.27 (2.41)
Field et al. [25]	RCT	40	39	12 weeks	4.4 (2.8)	4.3 (2.6)	4.4 (3.0)	4.4 (3.8)
Figueria et al. [26]	RCT	10	10	-	3.9 (1.10)	3.5 (1.10)	1.7 (1.00)	3.6 (1.10)
Haakstad and Bø [27]	RCT	29	32	-	OR	OR	OR	OR
Kordi et al. [28]	RCT	31	31	6 weeks	-	-	31.1 (17.59)	45.2 (14.57)
Martins et al. [29]	RCT	21	24	-	OR	OR	OR	OR
Miquelutti et al. [30]	RCT	78	71	-	4.7 (2.7)	4.5 (2.2)	5.1 (2.3)	4.8 (2.5)
Ozdemir et al. [31]	RCT	48	48	4 weeks	50.44 (26.92)	42.77 (26.57)	29.75 (23.84)	49.02 (24.89)
Petrov feiril et al. [32]	RCT	38	34	12 weeks	OR	OR	OR	OR
Sklempe et al. [33]	RCT	18	20	12 weeks	3 (2)	2 (2.1)	4.1 (2.1)	0.6 (0.7)
Sonmezer et al. [34]	RCT	20	20	8 weeks	43.60 (13.20)	41.80 (16.50)	17.20 (10.80)	38.40 (17.50)
Stafne et al. [35]	RCT	396	365	4 weeks	11.7 (14.9)	13.9 (17.7)	21.6 (22.0)	21.1 (21.1)
Yan et al. [36]	Non-RCT	45	44	12 weeks	-	-	7.67 (4.68)	9.61 (4.07)

Assessment of Risk of Bias

**Figure 3:**
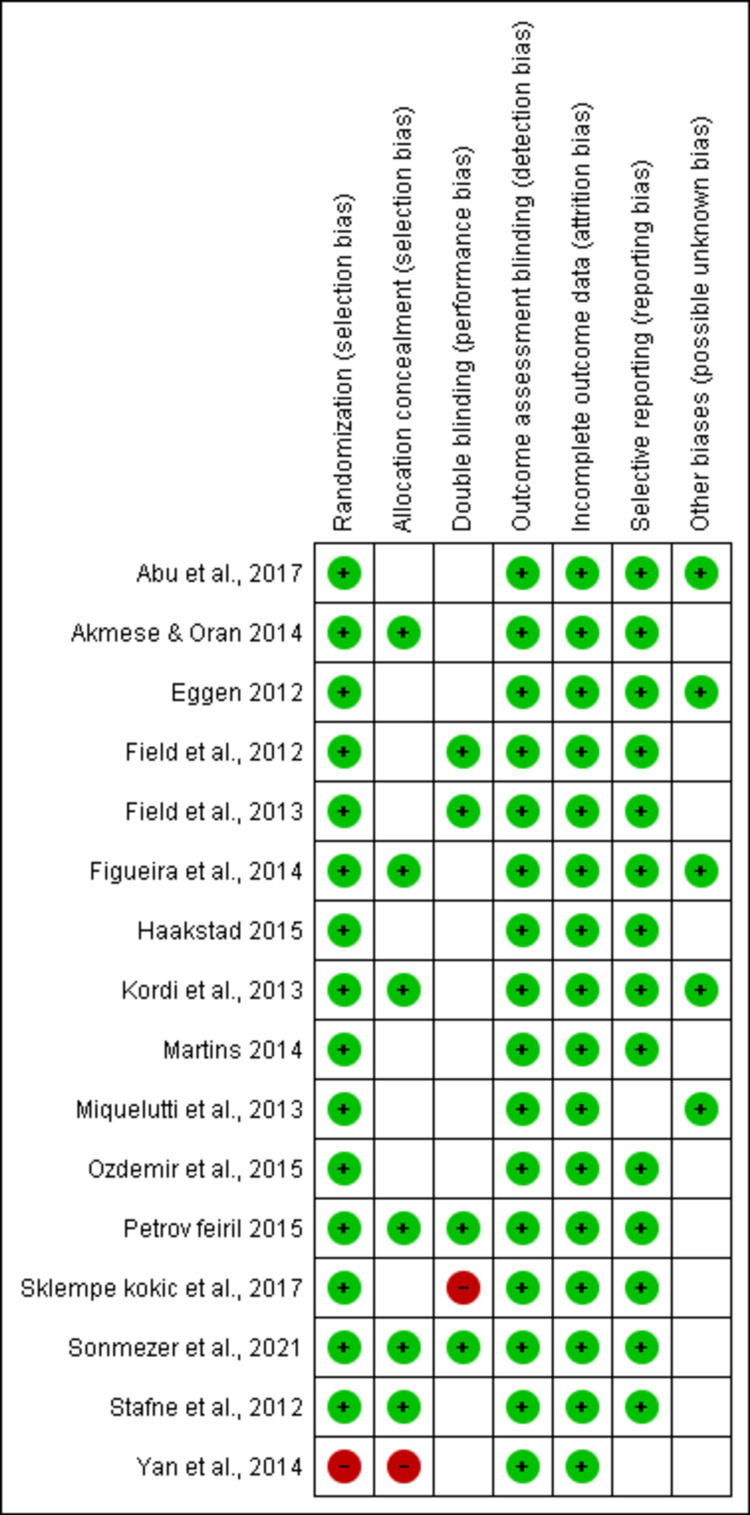
Risk of bias summary. Review of the author's judgment about each risk of bias item for each included comparison [19-34].

To rate the listed studies' methodological quality, Review Manager version 5.4 was utilized. The descriptions on the scale are as follows: "randomization (selection bias); concealment of allocation (selection bias); double blinding (performance bias); blinding of outcome assessment (detection bias); insufficient outcome data (attrition bias); selective reporting (reporting bias); and additional biases (possibly unknown bias)" are some examples of biases. Low-risk-of-bias, uncertain, and high-risk-of-bias studies were grouped together. When the included studies were assessed, it was discovered that all of them reported randomization except for one study conducted by Yan et al. [36], avoiding any potential selection bias. In six investigations, allocation concealment was clear; other studies were not clear. However, considering the nature of the test allocation, concealment is not possible. The included studies are of moderate quality (Figure 3).

Assessment of Methodological Quality

JADAD ratings are used to analyze each study's quality evaluation, which is detailed in Table 2. The study quality checklist elements were graded on a scale of 0 to 5, with a maximum of 5 points. Low-quality studies (0-2), medium-quality studies (3-4), and high-quality studies (5) were divided into these three categories. Out of the 16 studies considered, two had excellent quality and had a score of 5, seven had a score of 4, six had a score of 3, and one had a score of 1, indicating medium quality. In general, the research included in the analysis was of good caliber and contained trustworthy data.

**Table 2:** JADAD scores given by the authors for the studies included in the analysis. [21-36].

Study	Randomized	Double binding	Described appropriately?		Withdrawal and dropouts	Total score out of 5
			Randomization	Double blinding		
Abu et al. [21]	1	0	1	0	1	3
Akmese and Oran [22]	1	1	1	0	1	4
Eggen et al. [23]	1	0	1	0	1	4
Field et al. [24]	1	0	1	1	1	4
Field et al. [25]	1	0	1	1	1	4
Figueria et al. [26]	1	1	1	0	1	4
Haakstad et al. [27]	1	0	1	0	1	3
Kordi et al. [28]	1	1	1	0	1	4
Martins et al. [29]	1	0	1	0	1	3
Miquelutti et al. [30]	1	0	1	0	1	3
Odzemir et al. [31]	1	0	1	0	1	3
Petro feiril et al. [32]	1	1	1	1	1	5
Sklempe et al. [33]	1	0	1	0	1	3
Sonmezer et al. [34]	1	1	1	1	1	5
Stafne et al. [35]	1	1	1	0	1	4
Yan et al. [36]	0	0	0	0	1	1

The Severity of Symptoms for LBP and/or PGP During Pregnancy

**Figure 4:**
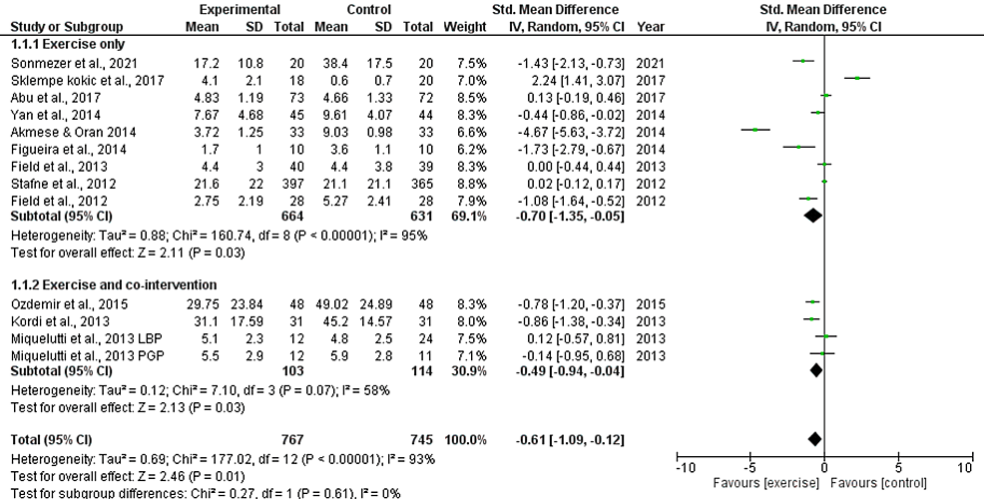
Forest plot of effects of exercise in comparison to a control group on the intensity of any sort of pregnancy-related pain (LBP, PGP) (RCTs). Studies using exercise-only treatments and those with exercise plus co-interventions both received sensitivity analysis. An analysis model with random effects was used [21,22,24-26,28,30,31,33-36].

There was "moderate" quality evidence from 12 RCTs (n=1512) [21,22,24-26,28,30,31,33-36] indicating a converse association between exercise and severity of any type of pain (LBP, PGP) during pregnancy. The pooled estimate was based on 12 RCTs (n=1512; SMD −0.61, 95% CI −1.09, −0.12, I2=93%) (Figure 4). The reduction was therapeutically notable but not statistically significant. The pooled estimate for the exercise-only group and the exercise + co-interventions pooled estimate did not vary substantially (P=0.61). In particular, there was "moderate" quality evidence from nine RCTs demonstrating that exercise-alone therapies decreased the severity of LBP during pregnancy (pooled estimate based on nine RCTs, n=1300; SMD 0.70, 95% CI 1.35, −0.05, I2=95%). The severity of either form of pain (LBP or PGP) during pregnancy was not reduced by exercise with co-interventions, according to moderate-quality data from three RCTs. The pooled estimate (n=217, SMD −0.49 95% CI 0.94, 0.04, I2=58%) was based on three RCTs.

Odds of LBP and/or PGP During Pregnancy

**Figure 5:**
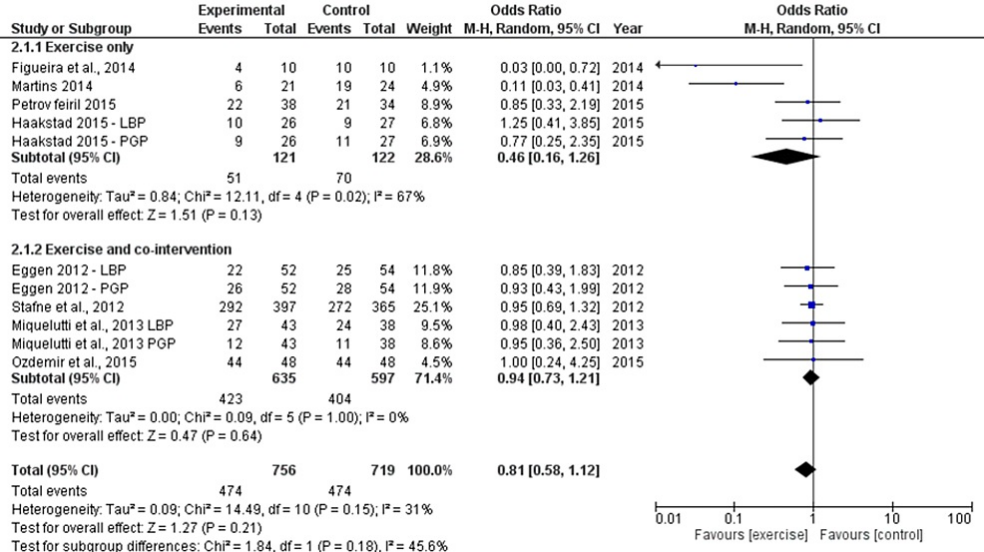
Forest plot of effects of exercise in comparison to a control group on the odds of any sort of pregnancy-related pain (LBP, PGP) (RCTs). Studies using exercise-only treatments and those with exercise plus co-interventions both received sensitivity analysis. An analysis model with random effects was used [23,26,27,29-32,35].

Eight RCTs (n=1232) [23,26,27,29-32,35] provided "moderate" quality evidence about the relationship between exercise and the odds of experiencing any kind of pain (LBP, PGP) during pregnancy. Due to the possibility of bias, the evidence's quality was reduced from "high" to "moderate." Compared to not exercising, exercise was not generally linked with a decreased likelihood of experiencing discomfort during pregnancy (pooled estimate based on 8 RCTs, n=1232; OR 0.81, 95% CI 0.58, 1.12, I2=31%) (Figure 4). The pooled estimates for the exercise-only treatments and the exercise + co-interventions estimates did not differ substantially (P=0.18). The odds of experiencing any kind of discomfort in the lumbopelvic area during pregnancy were not reduced by exercise-only therapies, according to "moderate" quality evidence (pooled estimate based on four trials, n=243; OR 0.46, 95% CI 0.16, 1.6; I2=67%) (Figure 5). Exercise, along with co-interventions, produced similar outcomes.

Discussion

The results of this meta-analysis, which included 15 controlled studies with 1885 participants, point to a moderate protective benefit of exercise against LBP and PGP in pregnancy. We used a focused search approach and implemented criteria for study design, the language of publication (English), and the year of publication, which may have inadvertently eliminated some older clinical studies. We only discovered a small number of RCTs that evaluated the impact of exercise on LBP and PGP, which reduced our ability to estimate the impacts on both outcomes. According to moderate-quality data, diverse exercise regimens (such as aerobic exercise, yoga, targeted strengthening exercises, generalized advancing exercises, or a mix of multiple types of exercises) did not lower the risk of developing LBP and PGP during pregnancy. Exercise, however, was an effective therapy to lessen the severity of LBP and PGP throughout pregnancy, and "poor" quality data from one RCT indicated that exercise during pregnancy lessened the severity of LBP.

Considering the effects of exercise on LBP in women who are not pregnant and the relatively few studies supporting its use in treating LBP during pregnancy, it is probable that exercise has a preventive effect against LBP. Exercise also seems to stop the recurrence of pain-related functional impairment [37,38]. In addition, a prospective cohort study of 2753 pregnant women found that, after adjusting for age, equalization, education, tobacco use, pre-pregnancy mass index, and LBP before this particular pregnancy, moderate to high levels of physical activity were linked to a 10% decrease in recurrences of LBP [39]. Once exercise becomes a routine, it appears to be more helpful in preventing future episodes of LBP and increases muscular endurance as well as muscular strength [40]. Physical activity done in a class or at work may have higher adherence rates, making it less likely that additional bouts of low back pain will occur [41]. The impact of exercise on low back pain may have been overestimated to the degree that women who were randomly assigned to exercise did not do so routinely and adherence to exercise was not adequate.

The findings support the idea that exercise is a way to lessen the degree of discomfort throughout pregnancy, despite the scant data. It should be emphasized that definitions for LBP and PGP were sometimes not made explicit by writers. Similarly, the methods employed to determine if symptoms were presently varied from self-reported to the objective evaluation carried out during clinical examination, which probably led to high levels of population variability or perhaps maybe incorrectly classified and incorrectly included women. The overall estimates are less confident due to the small number of studies that reported pain severity using outcomes intended to measure LBP-related impairment rather than pain. Furthermore, the significant levels of heterogeneity seen in the studies investigating the impact of exercise on the likelihood of pain comprising LBP and/or PGP (I2=95%) and on the severity of LBP (I2=67%) were perhaps caused by the characteristics of the women or the types of exercise used. Inevitably, subgroup analyses depending on the characteristics of the women, such as prior degree of exercise, pre-pregnancy BMI, or prior history of LBP, could not be carried out due to missing data.

At the conclusion of the exercise program utilized in a study conducted by Kluge et al. [42], it was found that pregnant women in the exercise group had lower pain intensity and higher functional ability compared to the control group. With the help of an individualized workout program and ongoing health education, Ozdmeir et al. [31] achieved comparable outcomes. They also came to the conclusion that all pregnant women with LBP may more readily adopt the counseling and exercise programs used in their study. Additionally, Morkved et al. [43] found that their exercise program inhibited the severity of LBP and PGP and enhanced their functional abilities in one of eight pregnant women at 36 weeks of pregnancy. Shim et al. [44] found that their exercise program reduced the magnitude of LBP and PGP for pregnant women but had no effect on their ability to work. These observations align with our findings, suggesting that exercise plays a crucial role in the management of LBP and PGP in pregnant women. According to Octaviani et al. [45], Pilates has demonstrated considerable benefits in reducing LBP, and these could constitute a research subject for future studies as they are beyond the scope of the current analysis. Similarly, Sonmezer et al. [34] revealed that an eight-week clinical Pilates exercise program reduces LBP and impairment in pregnant women more effectively than standard prenatal treatment with ergonomic education. Previous research demonstrated that significant pain and incapacity during pregnancy are related to inadequate muscle function in the lumbar and pelvic regions. Pilates workouts might therefore assure biomechanical recovery as a result of improved lumbopelvic stabilization [46].

Prevalence studies have shown that women tend to ignore the American College of Obstetricians and Gynecologists (ACOG) guidelines for physical activity, exercise less frequently when pregnant, and see a deterioration in their exercise routines as the pregnancy progresses [47]. This analysis conducted by Duncombe et al. [48] suggests that encouragement and positive reinforcement may be related to the higher energy expenditure that occurs from exercising when pregnant. Despite this, they continued to engage in an average amount of physical activity. The women in the control group who were not given any guidance or support exhibited a decrease in energy expenditure with the practice of physical exercise throughout pregnancy [49].

In the present analysis, the benefits of exercising for pregnancy-related LBP were established. Pregnant women in the exercise group stated that exercises improved their spinal alignment, confidence, capacity to carry out routine tasks, and comprehension of pregnancy and their bodily transformations. Since LBP is more common in pregnant women with a reported incidence of 57% to 69% [50] than it is in the population as a whole, the subject of LBP in pregnancy has sparked several studies in various fields [51]. Although exercise during pregnancy is seen favorably by healthcare professionals, much has already been accomplished to reduce its hazards. However, not every individual is mindful of or adhering to the most recent ACOG recommendations [52]. Therefore, it is critical to reevaluate sources that promote appropriate recommendations that are standardized and recommended by experts.

There are some significant limitations to the current meta-analysis that should be taken into account. The limitations of the study are as follows: (i) we acknowledge that using only a few trials raises the risk of selection inconsistency because our meta-analysis is based on a few RCTs, which can limit the generalizability and reliability of the findings. The results may be less robust as they are based on a smaller pool of studies. This limitation can lead to increased uncertainty and decreased confidence in the conclusions drawn from the meta-analysis. (ii) The final characteristics used in the chosen research were those that were documented. As a result, it is difficult to ascertain how the fundamental factors affected the meta-analysis's results. (iii) We did not look at associations among subgroups because of the limitations of the included studies. This limitation can hinder a comprehensive understanding of the intervention's effectiveness and its applicability to specific populations or settings. (iv) We also fell short of pinpointing evidence-based cutoff lines for clinically significant variations in study results. As a consequence, it is likely that the findings were overstated or understated in terms of their applicability.

## Conclusions

LBP represents one of the most typical musculoskeletal conditions among pregnant women. For some women, it could be an acute bout of persistent LBP and/or PGP, while for others, it might be excruciating pain during pregnancy. The present meta-analysis conclusively shows that exercise has a significant effect on LBP and PGP. However, a few side effects were unavoidable. Even though LBP may not be permanently cured, it can typically be successfully reduced. Accurate analysis and distinction between PGP and LBP are essential since there are variations in treatment. Suggested therapies include exercise, physiotherapy, stimulation of nerves, medical treatment, acupuncture, massage therapy, and yoga, depending on the situation. Further studies, such as randomized controlled trials and observational studies, are required to concentrate on these treatments in LBP management.
